# Health literacy strengths and needs among migrant communities from Portuguese-speaking African countries in Portugal: a cross-sectional study

**DOI:** 10.3389/fpubh.2024.1415588

**Published:** 2024-07-03

**Authors:** Ana Catarina Maia, Maria João Marques, Ana Rita Goes, Ana Gama, Richard Osborne, Sónia Dias

**Affiliations:** ^1^NOVA National School of Public Health, Public Health Research Centre, Comprehensive Health Research Center (CHRC), NOVA University Lisbon, Lisbon, Portugal; ^2^Health Sciences Research Unit: Nursing (UICISA: E), Nursing School of Coimbra (ESEnfC), Coimbra, Portugal; ^3^NOVA National School of Public Health, Public Health Research Centre, CHRC, REAL, NOVA University Lisbon, Lisbon, Portugal; ^4^Centre of Global Health and Equity, School of Health Sciences, Swinburne University of Technology, Hawthorn, VIC, Australia

**Keywords:** health literacy, health literacy questionnaire, health promotion, inequality, migrant health

## Abstract

**Introduction:**

Health literacy among migrants is a matter of public health and social justice. Migrants from diverse backgrounds encounter challenges such as linguistic barriers, cultural disparities, restricted access to health services, and heterogeneous migration statuses. Addressing these challenges requires careful consideration of their unique experiences and needs to promote equitable health outcomes. This can hinder their ability to navigate the healthcare system, understand health information, and engage in health-promoting behaviours. However, there is still a significant gap in our understanding of health literacy within migrant communities. This study has a dual aim: to identify health literacy strengths and needs among migrants from Portuguese-speaking African Countries (PALOP) countries in the Lisbon Metropolitan Area and to examine associations between demographic, socioeconomic, migration and health condition characteristics and the health literacy domains.

**Methods:**

A cross-sectional survey was conducted. Data were collected from 506 PALOP migrants using the Health Literacy Questionnaire (HLQ). We also collected demographic, socioeconomic, migration, and health condition data. We employed multiple linear regression to understand the relationship between the HLQ nine domains and these characteristics.

**Results:**

The HLQ scores revealed distinct patterns of health literacy between the groups. Health literacy needs were particularly evident in the domains related to feeling understood and supported by healthcare providers and navigating the healthcare system. Conversely, higher scores and potential strengths were observed in actively managing one’s health and understanding enough health information to make informed decisions. However, in these, the average scores suggest that a high proportion of people recognised difficulties. ‘The results also indicated that a higher educational level was associated with increased health literacy. In contrast, low self-perceived health status, living alone, shorter duration of residence in Portugal, and being either undocumented or in the process of obtaining legal status were associated with lower health literacy.

**Conclusion:**

Our study highlights the importance of migration-related variables and self-reported health status in understanding health literacy among migrant communities. Factors such as length of stay and low self-perceived health status are associated with potentially disadvantageous levels of health literacy, which could exacerbate health inequalities. Assessing these variables is critical to identify gaps in health literacy and develop tailored interventions to reduce health inequalities.

## 1 Introduction

In recent years, Europe has witnessed a significant rise in migration. As of 2022, it was estimated that approximately 27.3 million people residing in the European Union were non-EU citizens, accounting for 6.1% of the total population (1).

In Portugal, according to the Foreigners and Borders Service, in 2022, the number of foreign citizens with a residence permit was 781.915, an increase of 11.9% compared to 2022 (2).

Migrant populations are often concentrated in coastal regions and the Lisbon Metropolitan Area includes seven of the 10 municipalities with the most registered foreign citizens. The situation has also been observed for the Portuguese-speaking African countries (PALOP) migrant communities from Angola, Cape Verde, Guinea-Bissau, Mozambique and Sao Tome and Príncipe. Since the 1980s, PALOP migrant communities have steadily grown in Portugal, particularly in the Lisbon Metropolitan Area. By 2021, PALOP citizens with legal resident status numbered around 66.155 in this area, representing 74.92% of the national total (3).

Migration due to social and humanitarian crises is a concern and challenge for public health, given that the conditions in which immigration occurs significantly impact health.

The World Health Organization (WHO) 2010 established four principles for a public health approach to the health of migrants and host communities, namely: avoid disparities in health status and access to health services between migrants and the host population; ensure migrants’ health rights by limiting discrimination or stigmatisation of migrants and reducing difficulties in accessing health care; implement interventions that promote improved quality of life and reduce excess mortality and morbidity among migrant populations, and; minimise the negative impact of the migration process on migrant health outcomes (4). Health is thus an essential determinant of the successful integration of these populations into the host society. Ensuring good health and quality of life among migrant populations is critical for host countries (5).

Although studies conducted in Europe and Portugal indicate the “healthy migrant effect,” i.e., a tendency towards a self-perceived good health status on arrival in host countries, studies also suggest that during the time of stay, there is a decline in health status, namely an increase in the prevalence of chronic diseases and disability (6–8).

Indeed, migration is a profoundly human experience, involving individuals from diverse and often challenging backgrounds, each carrying unique health profiles and varying levels of health literacy (9, 10). This rich diversity profoundly affects the healthcare needs and outcomes of migrant populations (11). To truly address these needs, it is essential to understand and empathise with the multifaceted nature of their experiences (9). Recognising the complexity of these differences is essential for crafting effective public health interventions (12). Migrants come from diverse geographical, cultural and socioeconomic backgrounds, resulting in considerable variation in health status (13). Migrant’s health outcomes are influenced by many factors, including their country of origin, where differences in endemic diseases, health infrastructures and prevailing health behaviours play an important role in defining health profiles (9). In addition, the physical and psychological stress associated with the migration process can exacerbate existing health problems and introduce new health challenges (14). Socioeconomic status is another determinant, as limited access to essential resources such as nutritious food, stable housing and health services has a significant impact on overall health outcomes (11). Moreover, undocumented migrants often face significant barriers to accessing health care, mainly due to fear of deportation and ineligibility for services, which further jeopardises their health status (15).

Thus, it has been recognised that adopting strategies that mediate health promotion and prevention of chronic non-communicable diseases (NCDs) is one of the essential pillars of Public Health, especially for migrant populations (16, 17).

In this sense, health literacy has an important and crucial role in the health promotion of migrants in terms of equity of access to health care by migrant populations and the understanding and use of health information (16).

Health literacy is defined as the personal knowledge and skills that each person acquires in their daily activities, in social interactions, and from a generational perspective, mediated by organisational aspects and the availability of resources that enable people to access, understand, and use health information and services to promote their health and well-being (18, 19).

Indeed, health literacy enables people to make decisions and develop healthy behaviours that positively impact the management of their health (20). Studies suggest that low levels of health literacy are associated with poorer health outcomes, namely in terms of the prevalence of non-communicable diseases (NCDs), increased multimorbidity, health risk behaviours (e.g., sedentary lifestyle, poor eating habits, smoking), and limited capacity to engage in health promotion activities developed by health professionals and institutions (21, 22).

Understanding the health literacy strengths and needs of migrant communities is essential in reducing health inequities and promoting the health and well-being of these populations (13).

In the specific case of the migrant population, recent studies performed in the European context indicate decreased levels of health literacy in this population, leading to ineffective health management behaviours, poorer health status, and limited access to healthcare (10, 23). It has been shown that migrants encounter many challenges upon arrival in a new country.

Indeed, limited proficiency in the language of the host country can make it difficult to understand health information and access services (24). In turn, educational background, which significantly influences health literacy, varies widely among migrants (25). This variation can lead to diverse challenges in accessing and understanding health information, making it crucial to address educational differences in health communication strategies (26). Cultural beliefs and practices can also influence how health information is perceived and used (27). In addition, familiarity with the health care system in the country of origin may shape migrants’ expectations and interactions with health care providers in the host country (28). It is important to recognise that communication with health services and integration into the health system is one of the most critical issues. Previous experiences of the health system in a country of origin, cultural beliefs about the health-disease process, the significance attached to specific health initiatives such as screenings, and the stage of life cycle at which the migration process takes place can all influence this (29).

Addressing these disparities requires a tailored approach that considers the unique needs and circumstances of different migrant groups. Culturally competent care and targeted health literacy programmes are essential in bridging gaps and improving health outcomes for migrant populations (30).

However, in the Portuguese context, there is a lack of studies on health literacy in migrant populations from the PALOP countries, so this study expects to address this knowledge gap.

Thus, this study aims to: (1) identify the health literacy needs and strengths among migrants from PALOP countries living in the Lisbon Metropolitan Area using the Health Literacy Questionnaire; (2) examine the associations between the nine domains of health literacy and the demographic, socioeconomic, migratory and health condition characteristics of these migrant communities. Indeed, understanding these associations is crucial to identifying health literacy challenges and informing targeted interventions to improve health outcomes and reduce disparities. This knowledge will guide the development of effective health literacy support programmes, ultimately improving the well-being and integration of migrant populations.

## 2 Materials and methods

The study was part of a larger community-based co-design project in the Lisbon Metropolitan Area, entitled “Health Literacy, Health Promotion and Social Cohesion for the Prevention of NCDs among Migrant Populations” with migrant communities, using the Optimising Health Literacy and Access (Ophelia) process (31, 32). This study had a cross-sectional, descriptive, and analytical design conducted in the Lisbon Metropolitan Area between August and December 2020.

### 2.1 Study participants

The population studied is migrants from PALOP living in the Lisbon Metropolitan Area. We used the definition of migrant proposed by the International Organization of Migration (IOM), which refers to “*a person who moves from his or her usual place of residence, either within a country or across an international border, temporarily or permanently, and for a* var*iety of reasons*” (33).

The study covered a non-probability community-based sample, precisely a convenience sample, and the following eligibility criteria were defined: (a) being 18 years old or older; (b) being born in a PALOP country; (c) living in Portugal for less than 10 years; (d) living in the Lisbon Metropolitan Area, regardless of their migratory status; and (e) agreeing to participate voluntarily in the study.

The calculation of the sample size was based on the data available in the PORDATA (34). In 2019, it was estimated that there were approximately 68,000 migrants from the PALOP countries in the Lisbon metropolitan area, with the following distribution by country: 13552 people from Angola; 29,301 people from Cape Verde; 15,170 people from Guinea-Bissau; 2042 people from Mozambique; and 7,935 people from Sao Tome and Principe (34).

To determine the sample size, we considered a 95% confidence level, with a 5% margin of error, and the presence of 50% of the characteristics studied (i.e., assuming the worst-case scenario, since we do not know the size of the factors to be analysed), the estimated number of migrants indicated was at least 382 PALOP participants, with the following minimum proportions indicated by country of origin: 76 from Angola, 164 from Cape Verde, 85 from Guinea-Bissau, 12 from Mozambique and 45 from Sao Tome and Príncipe (35).

It is also important to mention that we used a sub-sample of a study of 1,126 migrant participants of different nationalities (PALOP, Brazil and Asia), developed with migrant communities as part of the above-mentioned project “Health Literacy, Health Promotion and Social Cohesion for the Prevention of NCDs among Migrant Populations.”

### 2.2 Data collection procedure

Participant recruitment involved the collaboration of governmental entities, non-governmental organisations, and organisations related to the migrant group, such as migrants’ associations, preserving the guidelines of the Helsinki Declaration (24) and the Guidance Note—Research on refugees, asylum seekers, and migrants of the European Commission (36).

Participants who met the eligibility criteria were invited to participate in the study. The data collection instruments were applied in Portuguese through interviews by trained researchers while they attended the organisations mentioned above. The informed consent form was obtained immediately before the participants completed the questionnaires, and anonymous coding of the questionnaires was performed. This study was approved by the Ethics Committee of NOVA Medical School (Ref.: 142/2019/CEFCM).

### 2.3 Instruments for data collection

Data were collected using the HLQ and a demographic, socioeconomic, migratory, and health condition characterisation questionnaire.

The HLQ has been widely used to assess individual or community health literacy needs and strengths (37–39). It consists of 44 items representing the nine health literacy domains (Table 1). The first five domains, composed of 23 items, cover the following: *1. “Feeling understood by healthcare providers,” 2. “Having sufficient information to manage my health,” 3. “Actively managing my health,” 4. “Having social health support,”* and *5. “Appraisal of health information.”* Each dimension is rated on a scale of 1 to 4, from “1-strongly disagree” to “4-strongly agree.” The last four domains, with 21 items, include *6. “Ability to actively engage with healthcare providers”*; *7. “Navigating the healthcare system”*; *8. “Ability to find good health information”*; and *9. “Understanding health information well enough to know what to do*. Each domain is rated on a scale of 1 to 5, from “1-cannot do or usually difficult” to “5- very easy.” The HLQ has robust psychometric properties, as demonstrated in its original English version (37, 40). Good psychometric properties have also been demonstrated in the Portuguese version of the HLQ, which was tested on a Portuguese population with Diabetes living in Lisbon (41). This version questionnaire was translated and adapted to Portuguese (41) according to the principles established by the authors of the HLQ (37, 42).

**Table 1:** Health literacy questionnaire (HLQ) scales with high and low descriptors of each construct (37).

Low level of the construct	High level of the construct
**1. Feeling understood and supported by healthcare providers**	
People who are low in this domain are unable to engage with doctors and other healthcare providers. They do not have a regular healthcare provider and/or have difficulty trusting healthcare providers as a source of information and/or advice.	Has an established relationship with at least one healthcare provider who knows them well and who they trust to provide useful advice and information and to assist them to understand information and make decisions about their health.
**2. Having sufficient information to manage my health**	
Feels that there are many gaps in their knowledge and that they do not have the information they need to live with and manage their health concerns.	Feels confident that they have all the information that they need to live with and manage their condition and to make decisions.
**3. Actively managing my health**	
People with low levels do not see their health as their responsibility, they are not engaged in their healthcare and regard healthcare as something that is done to them.	Recognise the importance and are able to take responsibility for their own health. They proactively engage in their own care and make their own decisions about their health. They make health a priority.
**4. Social support for health**	
Completely alone and unsupported for health.	A person’s social system provides them with all the support they want or need for health.
**5. Appraisal of health information**	
No matter how hard they try, they cannot understand most health information and get confused when there is conflicting information	Able to identify good information and reliable sources of information. They can resolve conflicting information by themselves or with help from others.
**6. Ability to actively engage with healthcare providers**	
Are passive in their approach to healthcare, inactive, i.e., they do not proactively seek or clarify information and advice and/or service options. They accept information without question. Unable to ask questions to get information or to clarify what they do not understand. They accept what is offered without seeking to ensure that it meets their needs. Feel unable to share concerns. The do not have a sense of agency in interactions with providers.	Is proactive about their health and feels in control in relationships with healthcare providers. Is able to seek advice from additional healthcare providers when necessary. They keep going until they get what they want. Empowered.
**7. Navigating the healthcare system**	
Unable to advocate on their own behalf and unable to find someone who can help them use the healthcare system to address their health needs. Do not look beyond obvious resources and have a limited understanding of what is available and what they are entitled to.	Able to find out about services and supports so they get all their needs met. Able to advocate on their own behalf at the system and service level.
**8. Ability to find good health information**	
Cannot access health information when required. Is dependent on others to offer information	Is an ‘information explorer’. Actively uses a diverse range of sources to find information and is up to date.
**9. Understanding health information well enough to know what to do**	
Has problems understanding any written health information or instructions about treatments or medications. Unable to read or write well enough to complete medical forms.	Is able to understand all written information (including numerical information) in relation to their health and able to write appropriately on forms where required.

The demographic, socioeconomic, migration and health condition questionnaire was based on the questions used in the 2014 National Health Survey carried out in Portugal (43). Demographic characteristics included age, gender, living arrangement, and country of origin. Socioeconomic status included educational level and monthly net income based on Portuguese the minimum national salary in 2020, which was around 635 euros per month (44). Migration background included different migration statuses, according to the IOM Glossary (21) definition for each of them, namely: (i) documented migrant—a migrant authorised to enter and to stay pursuant to the law of that State or to international agreements to which that State is a party and who is in possession of documents necessary to prove his or her regular status in the country; (ii) undocumented migrant—a non-national who enters or stays in a country without the appropriate documentation; and (iii) migrants in regularisation process—migrants in any process or programme by which the authorities of a State allow non-nationals in an irregular situation to stay lawfully in the country, by granting them a regular status; native language, and length of stay in Portugal. Health conditions included self-perceived health status, at least one non-communicable disease (NCD), and using health services in the last 12 months in Portugal. All categories and attributes of variables are represented in Supplementary Table S1.

### 2.4 Statistical analysis

Descriptive statistics were used to analyse demographic, socioeconomic, migration, and clinical data using frequencies, percentages, mean, and standard deviation.

The nine domains of the HLQ were characterised in terms of mean and standard deviation based on the HLQ User Manual scoring algorithm. HLQ missing values were replaced using the expectation maximisation algorithm described by Beauchamp et al. (45). Domains with four to five questions allowed two missing values to be imputed, and domains with six questions allowed three missing values to be imputed.

Linear regression models were used to assess the associations between sociodemographic, migration, and health factors and the nine domains of health literacy, with a multivariable-adjusted model (Enter Method) for independent variables under study, except for the “country of origin,” considering the scope of our research.

Regression coefficient (*b*) and their 95% confidence variables were individually estimated through simple/bivariate linear regression models for each covariate. Then, multivariable models included all independent variables using the enter method.

The models’ assumptions were analysed, verifying the residuals’ normality, homogeneity, and independence (46). All statistical analyses were performed using SPSS version 27 (47), and *p*-values <0.05 were considered statistically significant in the studies performed.

## 3 Results

### 3.1 Demographic, socioeconomic, migration and health characteristics

The study involved 506 migrants from PALOP countries who fulfilled the inclusion criteria and agreed to participate.

Demographic, socioeconomic, migratory and health characteristics are shown in Table 2.

**Table 2:** Demographic, socioeconomic, migratory and health condition characteristics of the study population (*N* = 506).

Variables	N	%(n)
Age Group*	506	
18–29 years		31.6(160)
30–39 years		30.4(154)
40–49 years		21.3(108)
50–59 years		11.3(57)
60–69 years		4.5(23)
> = 70 years		0.8(4)
*Missing*		0.0(0)
**Gender**	506	
Female		63.2(320)
Male		36.8(186)
Other		0 0.0(0)
*Missing*		0.0(0)
**Living Arrangement**	498	
Living Alone		19.6(99)
Living with others		78.6(399)
*Missing*		1.6(8)
**Country of birth**	506	
Angola		26.9(136)
Cape Verde		21.3(108)
Guinea-Bissau		26.1(132)
Mozambique		4.0(20)
São Tomé and Príncipe		21.7(110)
*Missing*		0.0(0)
**Educational level**	502	
0-9 years of education		45.8(232)
10–12 years of education		40.0(201)
More than 12 years of education		13.6(69)
*Missing*		0.8(4)
**Monthly net income**	492	
<650€		68.4(346)
> = 650€		28.9(146)
*Missing*		2.8(14)
**Length of stay in Portugal.**	505	
Less than one year		22.9(116)
Between 1 year and five years		62.8(318)
Between 6 years to 10 years		14.0(71)
*Missing*		0.2(1)
**Migration Status**	501	
Documented Migrant, under a co-operation agreement, refugee, asylum seeker		43.7(221)
Undocumented Migrant		13.6(69)
Regularisation process		41.7(211)
*Missing*		1.0(5)
**Native Language**	498	
Portuguese		64.6(327)
Creole		27.1(137)
Other (French, English, Native Dialect)		6.7(34)
*Missing*		1.6(8)
**Self-perceived health status**	499	
Very Good		16.2(82)
Good		170(33.6)
Fair		42.1(213)
Bad		5.9(30)
Very Bad		0.8(4)
*Missing*		1.4(7)
**At least one non-communicable disease (NCD)**	497	
No		62.5(316)
Yes		35.8(181)
*Missing*		1.8(9)
**Use of health services in the last 12 months**	463	
No		33.8(172)
Yes		67.2(340)
Primary health care		28.7(145)
Use of emergency services		22.7(115)
Other (private health services, welfare services)		7.7(39)
*Missing*		7.1(36)

^*^Mean age: 36.93 years, with an SD of 12.2.

The participants were mostly female (63.2%), were less than 45 years old, with a mean age of 36.93 years (SD = 12.2) and about 19.6% reported living alone. About 50% were from Angola (26.9%) and Guinea 26.1% followed by Sao Tome and Principe (21.7%), Cape Verde (21.3%) and Mozambique 4.0%. Most participants reported 0–9 years of education (45.8%), and 68.4% had a net monthly income lower than 650€. Many participants had resided in Portugal for over a year, less than 5 years (62.8%), and about 22.9% for less than a year. Most were documented migrants (43.7%), about 41.7% said they were in the process of regularisation, and 13.6% were undocumented. Almost 50% of the participants self-reported that their health was good/very good (49.8%) or fair/bad/very bad (48.8%). About 36.5% reported having at least one NCD. About 67.2% of the population has used health services in the last 12 months, with the majority using primary health care (28.7%).

### 3.2 HLQ scale scores

The distribution of the mean scores for the nine scales of the HLQ is shown in Table 3. In the present study, Cronbach’s alpha was above 0.80 for all scales, indicating a high level of internal consistency and reliability across the measurements used.

**Table 3:** Health literacy (HLQ) score scales (*N* = 506).

	Mean (SD) [95% IC]	Cronbachs alpha
Part 1-Range 1 (lowest) to 4 (highest)*		
**1. Feeling understood and supported by healthcare providers**	2.11 (0.84) [2.04–2.19]	0.84
**2. Having sufficient information to manage my health**	2.58 (0.58) [2.53–2.63]	0.83
**3. Actively managing my health**	2.95 (0.50) [2.91–3.00]	0.85
**4. Social support for health**	2.68 (0.66) [2.62–2.74]	0.85
**5. Appraisal of health information**	2.58 (0.56) [2.53–2.63]	0.84
Part 2-Range 1 (lowest) to 5 (highest)**		
**6. Ability to actively engage with healthcare providers**	3.38 (0.98) [3.29–3.46]	0.83
**7. Navigating the healthcare system**	3.03 (0.83) [2.95–3.10]	0.81
**8. Ability to find good health information**	3.36 (0.82) [3.29–3-43]	0.82
**9. Understanding health information well enough to know what to do**	3.63 (0.81) [3.56–3.70]	0.82

SD, Standard Deviation. CI, Confidence Interval.

*1 = strongly disagree, 2 = disagree, 3 = agree, 4 = strongly agree; **1 = cannot do or always difficult, 2 = usually difficult, 3 = sometimes difficult, 4 = usually easy, 5 = always easy.

For scales 1 to 5, the highest mean score was found for domain *3. “Actively managing my health”* (mean = 2.95, SD = 0.50), which suggested that most participants see their healthcare as their responsibility and agree to be able to engage in their healthcare. The lowest mean score was observed for dimension *1. “Feeling understood and supported by health care providers*” (mean = 2.11, SD = 0.84), indicating that most of the participants were unable to interact with healthcare professionals on a regular basis and agree that were difficult to trust them as advisors on health information. In addition, for scales *2. “Having sufficient information to manage my health”* (mean = 2.58; SD = 0.56) and *5. “Appraisal of health information”* (mean = 2.58, SD = 0.58), the scores indicated that half of the respondents felt that there were significant gaps in their knowledge and that they did not have the information they needed to manage their health problems and that, despite their best efforts, they could not understand most health information and were confused by conflicting information. In contrast, the other half of respondents felt the opposite.

For scales 6 to 9, the mean highest score was observed for domain *9. “Understanding health information well enough to know what to do”* (mean = 3.62; SD = 0.81), where most respondents reported that it was generally easy to understand all written information about their health and that they were able to write correctly on medical forms when required, but many still reported that it was sometimes difficult to. The mean lowest score was found for domain *7. “Navigating the healthcare system”* (mean = 3.03; SD = 0.83), which suggests that most respondents found it sometimes difficult to learn about available services and supports to meet all their needs and to advocate for themselves at the health system level. For scales *6.” Ability to actively engage with healthcare providers”* (mean = 3.38; SD = 0.98) and *8. “Ability to find good health information”* (mean = 3.36; SD = 0.81), on average, respondents answered that it was sometimes difficult to be proactive about their health, to ask questions to get information or to clarify what they do not understand and to have the ability to seek advice from other healthcare providers when necessary.

### 3.3 Health literacy associated factors

Table 4 shows the associations between health literacy scores and demographic, socioeconomic, migratory, and health condition characteristics.

**Table 4:** Associations between HLQ scales and demographic, socioeconomic, migration and clinical-related characteristics.

	1. Feeling understood and supported by healthcare providers			2. Having sufficient information to manage my health			3. Actively managing health		
	Model I^a^			Model I^a^			Model I^a^		
	*b*	SE	(95%,CI)	*b*	SE	(95%, CI)	*b*	SE	(95%, CI)
**Age**									
18–29 years (Ref.)	–	–	–	–	–	–	–	–	–
30–39 years	0.05	0.10	(−0.13, 0.24)	0.08	0.07	(−0.05, 0.22)	0.17*	**0.06**	**(0.05, 0.29)**
40–49 years	0.09	0.11	(−0.12, 0.30)	0.11	0.08	(−0.04,0.26)	0.20**	**0.07**	**(0.07, 0.34)**
50–59 years	−0.10	0.14	(−0.38, 0.18)	0.05	0.10	(−0.15,0.25)	0.10	0.09	(−0.08 0.28)
> = 60 years	0.13	0.20	(−0.26, 0.53)	0.21	0.14	(−0.08,0.49)	028*	**0.13**	**(0.03, 0.54)**
**Gender**									
Male (Ref.)	–	–	–	–	–	–	–	–	–
Female	0.01	0.08	(−0.15, 0.17)	−0.04	0.06	(−0.16, 0.08)	−0.12*	**0.05**	**(−0.22, −0.01)**
**Living alone**									
No (Ref.)	–	–	–	–	–	–	–	–	–
Yes	−0.16	0.10	(−0.35, 0.03)	−0.11	0.07	(−0.25, 0.02)	−0.02	0.06	(−0.14, 0.11)
**Educational level**									
0–9 years of education (Ref.)	–	–	–	–	–	–	–	–	–
10–12 years of education	−0.06	0.09	(−0.23, 0.11)	0.06	0.06	(−0.06, 0.19)	0.16	0.06	(0.05, 0.27)
More than 12 years of education	0.08	0.12	(−0.15, 0.31)	0.10	0.08	(−0.06, 0.27)	0.10	0.08	(−0.04, 0.25)
**Monthly Gros Income**									
>650€ (Ref.)	–	–	–	–	–	–	–	–	–
<=650€	−0.09	0.09	(−0.26, 0.08)	−0.08	0.06	(−0.20, 0.05)	0.00	0.06	(−0.11, 0.11)
**Migration Status**									
Documented (Ref.)	–	–	–	–	–	–	–	–	–
Undocumented	−0.20	0.12	(−0.43, 0.04)	−0.06	0.09	(0.51, −0.23)	0.02	0.08	(−0.14, 0.17)
In Regularisation Process	−0.26***	**0.09**	**(−0.43, −0.09)**	−0.10	0.06	(0.09–0.23)	−0.01	0.06	(−0.12, 0.10)
**Length of stay in Portugal**									
Between 6–10 years (Ref.)	–	–	–	–	–	–	–	–	–
Between 1–5 years	−0.37***	**0.12**	**(−0.61, −0.13)**	−0.07	0.09	(−0.24, 0.10)	0.14	0.08	(−0.02, 0.29)
Less than one year	−0.57***	**0.14**	**(−0.84, −0.29)**	−0.08	0.10	(−0.28, 0.12)	0.05	0.09	(−0.13, 0.22)
**Native language**									
Portuguese (Ref.)	–	–	–	–	–	–	–	–	–
Creole	0.03	0.08	(−0.13, 0.20)	−0.08	0.06	(−0.20, 0.04)	0.03	0.05	(−0.07, 0.14)
Other language (French, English, Dialecte)	−0.16	0.15	(−0.46, 0.14)	−0.05	0.11	(−0.27, 0.16)	0.01	0.10	(−0.19, 0.20)
**Self–perceived health status**									
Good/Very Good (Ref.)	–	–	–	–	–	–	–	–	–
Bad/Very Bad	−0.29***	**0.09**	**(−0.46, −0.11)**	−0.32***	**0.06**	**(−0.44, −0.20)**	−0.10	0.06	(−0.21, 0.01)
**At least one NCD**									
No (Ref.)	–	–	–	–	–	–	–	–	–
Yes	0.34***	**0.08**	**(0.18, 0.51)**	0.02	0.06	(−0.10, 0.14)	0.01	0.05	(−0.10, 0.12)
**Health care use last 12 months**									
No	−0.16	0.09	(−0.34, 0.02)	−0.03	0.07	(−0.17, 0.10)	0.08	0.06	(−0.04, 0.20)
Yes (Ref.)	–	–	–	–	–	–	–	–	–
*Adjusted R^2^*	*0.18*			*0.11*			*0.09*		
	4. Social support for health			5. Appraisal of health information			6. Ability to actively engage with healthcare providers				Model I^a^	Model I^a^	Model I^a^		*b*	SE	(95%, CI)	*b*	SE	(95%, CI)	*b*	SE	(95%, CI)
**Age**									
18–29 years (Ref.)	**–**	**–**	**–**	**–**	**–**	**–**	**–**	**–**	**–**
30–39 years	0.09	0.08	(0.06, 1.13)	0.09	0.07	(−0.04, 0.22)	0.01	0.11	(−0.22, 0.23)
40–49 years	−0.03	0.09	(−0.02, −0.36)	0.06	0.07	(−0.08, 0.21)	0.11	0.13	(−0.15, 0.36)
50–59 years	−0.05	0.12	(−0.02, −0.45)	−0.08	0.10	(−0.27, 0.11)	−0.13	0.17	(−0.46, 0.20)
> = 60 years	0.09	0.17	(0.03, 0.53)	−0.14	0.14	(−0.41, 0.13)	0.17	0.24	(−0.30, 0.65)
**Gender**									
Male (Ref.)	–	–	–	–	–	–	–	–	–
Female	0.05	0.07	(−0.08, 0.18)	−0.07	0.06	(−0.19, 0.04)	−0.07	0.10	(−0.26, 0.13)
**Living alone**									
No (Ref.)	–	–	–	–	–	–	–	–	–
Yes	−0.34***	**0.08**	**(−0.50, −0.19)**	−0.02	0.07	(−0.15, 0.11)	−0.08	0.12	(−0.31, 0.15)
**Educational level**									
0–9 years of education (Ref.)	–	–	–	–	–	–	–	–	–
10–12 years of education	0.14	0.07	(0.00, 0.28)	0.17**	**0.06**	**(0.05, 0.29)**	0.06	0.11	(−0.15, 0.26)
More than 12 years of education	0.11	0.10	(−0.09, 0.30)	0.31***	**0.08**	**(0.15, 0.47)**	−0.01	0.14	(−0.29, 0.27)
**Monthly Gros Income**									
>650€ (Ref.)	–	–	–	–	–	–	–	–	–
<=650€	−0.02	0.07	(−0.16, 0.12)	−0.07	0.06	(−0.19, 0.05)	0.01	0.10	(−0.19, 0.22)
**Migration Status**									
Documented (Ref.)	–	–	–	–	–	–	–	–	–
Undocumented	−0.08	0.10	(−0.28, 0.11)	−0.05	0.08	(−0.2, 0.11)	−0.11	0.15	(−0.40, 0.18)
In Regularisation Process	−0.08	0.07	(−0.22, 0.06)	−0.05	0.06	(−0.17, 0.06)	−0.10	0.10	(−0.31, 0.10)
**Length of stay in Portugal**									
Between 6–10 years (Ref.)	–	–	–	–	–	–	–	–	–
Between 1–5 years	0.05	0.10	(−0.14, 0.24)	0.05	0.08	(−0.12, 0.21)	−0.05	0.14	(−0.33, 0.24)
Less than one year	−0.36	0.10	(−0.36, 0.10)	0.05	0.10	(−0.14, 0.24)	−0.32	0.17	(−0.65, 0.02)
**Native language**									
Portuguese (Ref.)	–	–	–	–	–	–	–	–	–
Creole	−0.08	0.07	(−0.22, 0.06)	−0.04	0.06	(−0.16, 0.07)	0.06	0.10	(−0.14, 0.26)
Other language (French, English, Dialecte)	−0.22	0.13	(−0.47, 0.03)	0.12	0.11	(−0.09, 0.33)	−0.54***	**0.19**	**(−0.90, −0.17)**
**Self–perceived health status**									
Good/Very Good (Ref.)	**–**	**–**	**–**	**–**	**–**	**–**	**–**	**–**	**–**
Bad/Very Bad	−0.18*	**0.07**	**(−0.32, −0.04)**	−0.12	0.06	(−0.23, 0.00)	−0.42***	**0.10**	**(−0.63, −0.22)**
At least one NCD									
No (Ref.)	–	–	–	–	–	–	–	–	–
Yes	0.05	0.07	(−0.09, 0.18)	0.06	0.06	(−0.06, 0.17)	−0.03	0.10	(−0.23, 0.17)
**Health care use last 12 months**									
No	0.03	0.08	(−0.12, 0.18)	−0.13*	**0.06**	**(−0.26, −0.01)**	−0.44***	**0.11**	**(−0.66, −0.22)**
Yes (Ref.)	**–**	**–**	**–**	**–**	**–**	**–**	**–**	**–**	**–**
*Adjusted R^2^*	*0.12*			*0.11*			*0.13*		
	7. Navigating the health care system			8. Ability to find good health information			9. Understanding health information well enough to know what to do				Model I^a^	Model I^a^	Model I^a^		*b*	SE	(95%, CI)	*b*	SE	(95%, CI)	*b*	SE	(95%, CI)
**Age**									
18–29 years (Ref.)	**–**	**–**	**–**	**–**	**–**	**–**	**–**	**–**	**–**
30–39 years	0.09	0.10	(−0.10, 0.28)	0.10	0.09	(−0.08, 0.28)	0.09	0.09	(−0.09, 0.27)
40–49 years	0.15	0.11	(−0.06, 0.36)	0.02	0.10	(−0.18, 0.22)	0.20	0.10	(0.00, 0.40)
50–59 years	−0.05	0.14	(−0.33, 0.23)	−0.21	0.13	(−0.48, 0.05)	−0.16	0.13	(−0.43, 0.10)
> = 60 years	0.18	0.20	(−0.23, 0.58)	−0.50**	0.18	(−0.85, −0.15)	−0.18	0.19	(−0.56, 0.20)
**Gender**									
Male (Ref.)	**–**	**–**	**–**	**–**	**–**	**–**	**–**	**–**	**–**
Female	−0.01	0.08	(−0.17, 0.16)	−0.05	0.08	(−0.2, 0.11)	−0.08	0.08	(−0.23, 0.08)
**Living alone**									
No (Ref.)	**–**	**–**	**–**	**–**	**–**	**–**	**–**	**–**	**–**
Yes	−0.05	0.10	(−0.24, 0.14)	−0.05	0.09	(−0.23, 0.13)	−0.01	0.09	(−0.19, 0.17)
**Educational level**									
0–9 years of education (Ref.)	**–**	**–**	**–**	**–**	**–**	**–**	**–**	**–**	**–**
10–12 years of education	0.12	0.09	(−0.05, 0.30)	0.30***	**0.08**	**(0.13, 0.46)**	0.34***	**0.08**	**(0.18, 0.51)**
More than 12 years of education	0.14	0.12	(−0.09, 0.38)	0.43***	**0.11**	**(0.21, 0.65)**	0.29**	**0.11**	**(0.07, 0.52)**
**Monthly Gros Income**									
>650€ (Ref.)	**–**	**–**	**–**	**–**	**–**	**–**	**–**	**–**	**–**
<=650€	−0.08	0.09	(−0.25, 0.09)	−0.05	0.08	(−0.21, 0.11)	0.03	0.08	(−0.13, 0.20)
**Migration Status**									
Documented (Ref.)	**–**	**–**	**–**	**–**	**–**	**–**	**–**	**–**	**–**
Undocumented	−0.39***	**0.12**	**(−0.63, −0.15)**	−0.08	0.12	(−0.30, 0.15)	−0.09	0.12	(−0.32, 0.14)
In Regularisation Process	−0.16	0.09	(−0.33, 0.02)	−0.07	0.08	(−0.24, 0.09)	−0.09	0.08	(−0.32, 0.14)
**Length of stay in Portugal**									
Between 6–10 years (Ref.)	**–**	**–**	**–**	**–**	**–**	**–**	**–**	**–**	**–**
Between 1–5 years	−0.25*	**0.12**	**(−0.49, −0.01)**	0.03	0.11	(−0.20, 0.25)	0.08	0.11	(−0.15, 0.30)
Less than one year	−0.40**	**0.14**	**(−0.68, −0.12)**	−0.08	0.13	(−0.35, 0.18)	−0.03	0.13	(−0.30, 0.23)
**Native language**									
Portuguese (Ref.)	**–**	**–**	**–**	**–**	**–**	**–**	**–**	**–**	**–**
Creole	−0.14	0.09	(−0.31, 0.03)	−0.17*	**0.08**	**(−0.33, −0.01)**	−0.07	0.08	(−0.23, 0.08)
Other language (French, English, Dialecte)	**−**0.24	0.16	(−0.55, 0.06)	−0.07	0.15	(−0.36, 0.22)	−0.26	0.15	(−0.55, 0.03)
**Self–perceived health status**									
Good/Very Good (Ref.)	**–**	**–**	**–**	**–**	**–**	**–**	**–**	**–**	**–**
Bad/Very Bad	−0.39***	**0.09**	**(−0.56, −0.21)**	−0.30***	**0.08**	**(−0.46, −0.13)**	−0.32***	**0.08**	**(−0.49, −0.16)**
**At least one NCD**									
No (Ref.)	**–**	**–**	**–**	**–**	**–**	**–**	**–**	**–**	**–**
Yes	0.07	0.09	(−0.10, 0.24)	0.10	0.08	(−0.06, 0.26)	0.18*	**0.08**	**(0.02, 0.34)**
**Health care use last 12 months**									
No	−0.10	0.09	(−0.28, 0.09)	−0.02	0.09	(−0.19, 0.15)	−0.18*	**0.09**	**(−0.36, −0.01)**
Yes (Ref.)	**–**	**–**	**–**	–	–	–	–	–	–
*Adjusted R^2^*	*0.11*			*0.18*			*0.12*		

^a^Adjusted for all variables. *p-value < 0.05; **p-value < 0.01, ***p-value < 0.001. Bold values indicate significant *p*-value.

The findings showed that being aged between 40 and 49 (*b* = 0.20, 95%CI 0.07 to 0.34) and between 60 and 69 (*b* = 0.28, 95%CI 0.03 to 0.25) was associated with higher health literacy scores in domain *3. “Actively managing my health”* while being aged > = 70 (*b* = −1.11, 95%CI −1.88 to −0.35) was associated with lower health literacy scores in domain *8. “Ability to find good health information.”*

In turn, being female (*b* = −0.12, 95%CI −0.22 to −0.01) was associated with lower health literacy scores in domain *3. “Actively managing my health”* and living alone (*b* = −0.34, 95%CI −0.50 to −0.19) were associated with lower scores in domain *4. “Social support for health.”*

Educational levels of 10–12 years were associated with higher scores in domains *5. “Appraise health information”* (*b* = 0.17, 95%CI 0.05 to 0.29), *8. “Ability to find good health information”* (*b* = 0.30, 95%CI 0.13 to 0.46) and *9. “Ability to understand health information well enough to know what to do”* (*b* = 0.34, 95%CI 0.18 to 0.51). Similarly, having more than 12 years of schooling was associated with domains *5. “Appraise health information”* (*b* = 0.31, 95%CI 0.15 to 0.47), *8. “Ability to find good health information”* (*b* = 0.43, 95%CI 0.21 to 0.65) and *9. “Ability to understand health information well enough to know what to do”* (*b* = 0.29, 95%CI 0.07 to 0.52).

Considering migration status, being an undocumented migrant (*b* = −0.39, 95%CI -0.63 to −0.15) was associated with lower levels in domain *7. “Navigating the health care system”* and being in the process of regularisation were associated with lower scores in domain *1.” Feeling understood and supported by healthcare providers.”*

Living in Portugal for between 1 and 5 years was associated with lower levels in domains *1*. *“Feeling understood and supported by healthcare providers”* (*b* = −0.37, 95%CI −0.61 to −0.13) and *7. “Navigating the healthcare system”* (*b* = −0.25, 95% −0.49 to −0.01).

Lower scores for time of residence of less than 1 year were observed in domains *1. “Feeling understood and supported by healthcare providers”* (*b = −0.57, 95% CI* −*0.84 to − 0.29*), *and 7, “Navigating the healthcare system”* (*b* = −0.40; 95% CI −0.68 and − 0.12).

Having Creole as a native language was associated with lower scores in domain *8. “Ability to find good health information”* (*b* = −0.17, 95%CI −0.33, 0.01) while having another language as a native language was associated with lower scores in domain *6—*“*Ability to actively engage with healthcare providers”* (*b* = −0.54, 95%CI −0.90 to 0.17).

Self-perceived health status as “Fair/Bad/ Very Bad” was associated with lower health literacy scores in the domains *1.” Feeling understood and supported by healthcare providers”* (*b* = −0.29, 95%CI −0.46 to −0.11); *2. “Having sufficient information to manage my health”* (*b* = −0.32, 95%CI −0.44 to −0.22); *4. “Social support for health”* (*b* = −0.18, 95%CI −0.32 to 0. 04); *6—“Ability to actively engage with healthcare providers”* (*b* = −0.42, 95%CI −0.63 to −0.22); *7. “Navigating the healthcare system”* (*b* = −0.39, 95%CI −0.56 to −0.21); *8. “Ability to find good health information”* (*b* = −0.30, 95%CI −0.46 to −0.13); and *9. “Understanding health information well enough to know what to do”* (*b* = −0.32, 95%CI −0.36 to −0.01). The presence of at least one NCD was associated with higher scores in domains *1. “Feeling understood and supported by healthcare providers”* (*b* = 0.34, 95%CI 0.18 to 0.51) and *9. “Understanding health information well enough to know what to do”* (*b* = 0.18, 95%CI 0.02 to −0.34). In turn, not using healthcare in the last 12 months was associated with lower scores in domains *5.” Appraisal of health information”* (*b* = −0.13, 95%CI −0.26 to −0.01); *6. “Ability to actively engage with healthcare providers”* (*b* = −0.44, 95%CI −0.66 to −0.22) and *9. “Understanding health information well enough to know what to do”* (*b* = −0.18, 95%CI −0.36 to −0.01).

## 4 Discussion

The findings of our study highlight the diverse health literacy challenges faced by PALOP migrants.

Overall, PALOP migrants demonstrate specific health literacy needs, particularly concerning their interactions with healthcare professionals and systems and their capacity to identify and value high-quality health information. In turn, the potential health literacy strengths of PALOP migrants seem to be more evident in the domain related to their active health management.

Notably, PALOP migrants exhibit strengths in actively managing their health, but their scores in most other domains HLQ are lower compared to those of the general Portuguese population with Diabetes Mellitus (41). These findings can be followed by the difficulty that PALOP migrants face in the context of integration in Portugal in accessing health services, which consequently leads to the establishment of an unsatisfactory therapeutic relationship with health professionals due to the lack of regular contact with them.

Our findings suggest substantial diversity in health literacy and its determinants among PALOP migrants.

Specifically, a shorter residence duration in Portugal and undocumented status are associated with lower health literacy. This association often manifests as feelings of being misunderstood and unsupported by healthcare professionals, reduced engagement with these professionals, and difficulties in utilising health information and accessing health services.

Consistent with prior research, our results indicate that migrant populations with shorter residency periods exhibit lower health literacy, particularly in areas involving interactions with health professionals, health services, and proactive health management (45, 48, 49). Factors such as limited access to health services, language barriers, cultural differences, low education levels, social stigma, and complex interactions with health professionals significantly contribute to this (29, 50–52). Additionally, undocumented migrants with shorter stays often face compounded challenges, making it difficult to establish a stable healthcare routine and develop health literacy over time. However, it is essential to recognise that migrants with shorter stays are often undocumented, complicating healthcare access and continuity of care, thereby hindering the development of health literacy over time.

Language barriers present another substantial challenge for PALOP migrants. Those who speak Creole or other languages face significant difficulties in navigating the healthcare system and accessing health information. This is consistent with previous studies showing that migrants with a native language different from that of the host country often face language barriers in health services (45, 53). A similar situation is evident in Portugal, as highlighted in previous studies, where migrant PALOP populations face language difficulties when accessing health services (50, 54). Even in official Portuguese-speaking contexts, linguistic diversity, including fluency in Creole and other dialect can pose significant challenges for migrants. Urgent measures must be implemented to ensure that migrants can access the health services they require.

Our study findings, which reveal a significant association between negative self-perception of health status and lower health literacy scores in almost all domains, underscore the crucial health literacy needs in the PALOP migrant community. Supported by previous evidence, our results suggest that migrants with low self-perceived health status may encounter challenges in self-management, understanding the health-disease process, health-seeking behaviour, and self-efficacy (37, 38, 40), and therefore may negatively impact good health literacy.

Self-perceived health status is a crucial indicator of health outcomes and mortality, especially among disadvantaged groups like migrants and ethnic minorities (55, 68). Studies show that the longer migrants stay in host countries, the worse they perceive their health compared to when they arrived, as seen in the Portuguese (7, 50). Thus, our study emphasises the importance of identifying the main difficulties related to health literacy experienced by PALOP migrants with a low self-perception of their health status, so that they can better find and evaluate health information. This will enable them to make informed decisions about their health and interact proactively with professionals and others.

On the other hand, in our study, the presence of at least one NCD among PALOP migrants seems to improve better health literacy in aspects related to relationships with health professionals and understanding health information.

However, research on the link between migrants with NCDs and their health literacy is inconsistent. For instance, Wångdahl et al.’s (56) study in Sweden found that chronic diseases were linked to lower functional health literacy but not to comprehensive health literacy. Conversely, Berens et al. (25) observed lower health literacy scores in Germany when only chronic diseases were considered. Meanwhile, Rheault et al.’s research in Australia among indigenous populations identified chronic illness as a predictor of higher health literacy in ability to access, understand, and use health-related information (57). Chronic diseases can increase health literacy (69), but this is uncertain for migrants due to language barriers and cultural differences, so the findings of our study emphasise the need for more research into the impact of these factors on migrant’s health literacy (25, 56, 57).

According to our study, lower health literacy is linked to the non-use of health services among migrants from PALOP in the past year. This is particularly evident in evaluating health information, communicating with health professionals, and managing health problems, posing significant challenges. The factors contributing to low health literacy are complex. Lower health service use by migrants, compared to non-migrants, may indicate better overall health, access barriers, or personal and cultural preferences (50, 66, 67). For PALOP migrants, who often rely on healthcare professionals as critical sources of information, particularly about navigating healthcare in Portugal, not using health services can hinder access to vital health information (58). This situation potentially affects the ability to form culturally sensitive therapeutic relationships and manage health effectively, which often depends on active support from healthcare providers.

Our study also reveals that people over 60 often need more information to find quality health resources, a similar pattern observed in other studies involving older migrants (10, 48). However, our results, also show that these older migrants manage their health better than people aged between 30 and 49. Indeed, research about ageing and health literacy in Portugal presents contradictory results. Some studies indicate a negative correlation in older diabetes patients (41) while others suggest positive associations (59, 60). These differences highlight the dual effects of the ageing process, which can lead to different health literacy needs and resources: greater involvement in healthcare due to chronic illnesses can improve management skills, but cognitive decline can limit the ability to understand health information (10, 41, 48, 59, 60).

Our study suggests that migrant women have lower health literacy, particularly in active health management. This finding is consistent with previous research examining health service use and the ability to locate health information (48, 61). This decline in health literacy may be due to cultural factors, particularly the social roles of African women, who often prioritise family responsibilities over managing their own health, which can lead to neglect of their personal health, particularly in relation to mental health and sexual and reproductive health (54). Future research should therefore explore the intersection of migration, gender and health literacy to develop strategies to improve health outcomes and reduce inequalities among migrant women.

An important aspect to highlight in our study is that migrants living alone appear to be more demanding in terms of health support. This finding is consistent with what has been observed in groups of people with chronic diseases such as diabetes, rheumatic diseases (62). However, no other studies have shown an association between migrant household composition and health literacy, and this is important for future research. Living alone may lead to less use of social and family networks, which have been identified as a critical aspect of health literacy, as these networks facilitate greater health care and health information seeking and decision making (63, 64).

In our study, a higher level of education among PALOP migrants is also one of the factors strongly associated with health literacy, especially in terms of greater recognition of and involvement in self-management of health and more excellent knowledge and understanding of how to use health services and health information. These aspects are in line with previous studies carried out with migrant populations (36, 37, 39, 40), which highlight the need to address how this population interacts with the health system, how they establish an engagement and therapeutic relationship with health professionals, and how they understand and use health information in their daily lives to make health decisions.

A critical aspect of our study is that no significant associations were found between monthly net income and the nine health literacy domains. These results are consistent with other studies of migrant populations, such as in Spain with people from North Africa (65) and in Australia with Chinese migrants (48), where no significant associations were found. However, given the well-known impact of socioeconomic factors on the social gradient of health literacy, it is important that the results of our study are interpreted carefully, and that further research is conducted in this area.

Drawing from the nuanced findings of our study on health literacy among PALOP migrants in Portugal, a holistic and inclusive approach to health policy and practice is urgently needed. The challenges identified, such as shorter length of stay, undocumented status, language barriers, poor perception of health, limited use to health services, cultural differences, and—underscore the necessity for targeted actions that address these specific health literacy needs.

For instance, migrants with a shorter length of stay often lack familiarity with the healthcare system, making it difficult for them to access necessary services. Being undocumented further complicates their situation by restricting access to health services and increasing their vulnerability. Language barriers can lead to misunderstandings and inadequate care, while poor perception of health might discourage seeking care. Limited access to health services, compounded by systemic barriers, exacerbates health inequalities. Additionally, cultural differences can isolate migrants, making them hesitant to seek help.

Meanwhile, the demonstrated capacity of these migrants to actively manage their health highlights a foundation of strengths upon which interventions can be built. Healthcare professionals, policymakers, and the broader community must collaborate to create a more accessible and welcoming health environment. This collective effort should aim to remove existing barriers and leverage the health literacy potentials of these communities. By doing so, we can ensure that all individuals, regardless of their migration status, can achieve the highest possible standard of health and well-being. This inclusive approach not only benefits the migrants but also strengthens the overall health system by promoting equity, diversity, and social cohesion.

### 4.1 Limitations and strengths of the study

The current study has certain limitations that need to be considered. Firstly, due to its cross-sectional design, it is not possible to establish causality relationships. Secondly, the sample used was non-probabilistic, so it is important to be cautious when generalising the results. In addition, a significant limitation of this study is the use of convenience sampling to select participants. Since individuals were chosen based on availability and convenience, the results may not be generalizable to the broader population due to selection bias. Besides the above-mentioned limitations, the sample of this study did not meet the minimum number of participants per country, which could affect the generalisability of the findings across cultures and geographies. It is therefore important to interpret the results with caution, considering this limitation.

Finally, even though Cronbach’s alpha demonstrated good internal consistency across all scales of the questionnaire in the study sample, it is worth noting that cultural validation of the HLQ among migrants from PALOP was not conducted.

Our study has several strengths. It is one of the first studies conducted in Portugal to assess the health literacy of the PALOP migrant population; therefore, it is an important study for a better understanding of health literacy in this population. The PALOP migrant population has a history of continuous migration since the 1980s, making this study even more relevant. Additionally, the questionnaires were administered with the support of the research team, which made it possible for any doubts that may have arisen to be clarified. This ensured the accuracy of the data collected.

## 5 Conclusion

Our study has allowed us to identify the health literacy needs of PALOP migrants in Portugal, particularly in engagement with health professionals, interaction with the health system and the ability to appraise health information. These findings are particularly relevant for those with a lower self-perceived health status and a shorter period of residence in Portugal. Our study has allowed an intersectional analysis from the perspective of demographic, socioeconomic, migratory, and clinical factors. It also shows that within a group of migrants, subgroups with different profiles, including diverse strengths and needs, can provide valuable information for developing interventions.

Finally, given the continuous increase in migratory flows from PALOP countries to Portugal, the results of our study inform the need for an unquestionable approach from a public health perspective. The implementation of integration strategies, in terms of health promotion, among these populations is fundamental in our country’s first years of residence, especially given the observation of a low self-perceived health status.

Therefore, it is necessary to strengthen the sense of equity in health policies and the adoption of interventions by health organisations. It will address a better increase in health literacy, making the health system more flexible and health information more accessible and understandable, allowing them to make effective health decisions and actively participate in the therapeutic relationship with health professionals.

## Data availability statement

The raw data supporting the conclusions of this article will be made available by the authors, without undue reservation.

## Ethics statement

The studies involving humans were approved by Ethics Committee of NOVA Medical School (Ref.: 142/2019/CEFCM). The studies were conducted in accordance with the local legislation and institutional requirements. Written informed consent for participation in this study was provided by the participants’.

## Author contributions

AM: Conceptualization, Data curation, Formal analysis, Funding acquisition, Investigation, Methodology, Project administration, Resources, Validation, Writing – original draft, Writing – review & editing. MM: Data curation, Funding acquisition, Project administration, Writing – review & editing. ARG: Conceptualization, Methodology, Project administration, Validation, Writing – review & editing. AG: Funding acquisition, Project administration, Writing – review & editing. RO: Conceptualization, Methodology, Validation, Writing – review & editing. SD: Conceptualization, Methodology, Project administration, Validation, Writing – review & editing.
