# Supplementary material for: Health literacy strengths and needs among migrant communities from Portuguese-speaking African countries in Portugal: a cross-sectional study
Source: Front Public Health. 2024 Jul 3;12:1415588. doi: 10.3389/fpubh.2024.1415588 (PMC11253791; doi:10.3389/fpubh.2024.1415588)
Supplement: Supplementary file 1 [file Table_1.DOCX]

Supplementary Material

# Supplementary Table

# Table S1- Variables under study characterisation (Supplementary Files)

| **Variables** | **Categories** |
| --- | --- |
| Age groups | 18-29 years; 30-39 years; 40-49 years; 50-59 years; 60-69 years^a^; >= 70 years^a^ |
| Gender | Female; Male; Other |
| Living Arrangement | Living alone; Living with others |
| Country of Origin | Angola; Cape Verde; Guinea-Bissau; Mozambique; Sao Tome and Principe |
| Educational level | 0-9 years of education; 10-12 years of education; More than 12 years of education |
| Monthly net income | <650€; >=650€ |
| Length of stay in Portugal | Less than one year; Between 1 year to 5 years; Between 6 years to 10 years |
| Migration Status | Documented migrant, under a co-operation agreement, refugee, asylum seeker; Undocumented Migrant; Migrant in a regularisation process |
| Native Language | Portuguese; Creole; Other (French, English, Native Dialect) |
| Self-perceived health status | Very Good^b^, Good^b^; Fair^c^; Bad^c^; Very Bad^c^ |
| At least one non-communicable disease (NCD) | No; Yes |
| Use of health services in the last 12 months | No; Yes (If “Yes” Primary health care; Use of emergency services; Other (private health services, welfare services) |

Note: ^a^ In Multivariable Regression Models variables were merged due to the low frequencies.

^b^ In Multivariable Regression Models variables were merged.

^c^ In Multivariable Regression Models, variables were merged due to the low frequencies of the “bad” and “very bad” categories
